# CaMKII Binding to GluN2B Is Differentially Affected by Macromolecular Crowding Reagents

**DOI:** 10.1371/journal.pone.0096522

**Published:** 2014-05-05

**Authors:** Dayton J. Goodell, Tatiana A. Eliseeva, Steven J. Coultrap, K. Ulrich Bayer

**Affiliations:** Department of Pharmacology and Neuroscience Program, University of Colorado Denver, School of Medicine, Aurora, Colorado, United States of America; University of Alabama at Birmingham, United States of America

## Abstract

Binding of the Ca^2+^/calmodulin(CaM)-dependent protein kinase II (CaMKII) to the NMDA-type glutamate receptor (NMDAR) subunit GluN2B controls long-term potentiation (LTP), a form of synaptic plasticity thought to underlie learning and memory. Regulation of this interaction is well-studied biochemically, but not under conditions that mimic the macromolecular crowding found within cells. Notably, previous molecular crowding experiments with lysozyme indicated an effect on the CaMKII holoenzyme conformation. Here, we found that the effect of molecular crowding on Ca^2+^/CaM-induced CaMKII binding to immobilized GluN2B *in vitro* depended on the specific crowding reagent. While binding was reduced by lysozyme, it was enhanced by BSA. The ATP content in the BSA preparation caused CaMKII autophosphorylation at T286 during the binding reaction; however, enhanced binding was also observed when autophosphorylation was blocked. Importantly, the positive regulation by nucleotide and BSA (as well as other macromolecular crowding reagents) did not alleviate the requirement for CaMKII stimulation to induce GluN2B binding. The differential effect of lysozyme (14 kDa) and BSA (66 kDa) was not due to size difference, as both dextran-10 and dextran-70 enhanced binding. By contrast, crowding with immunoglobulin G (IgG) reduced binding. Notably, lysozyme and IgG but not BSA directly bound to Ca^2+^/CaM in an overlay assay, suggesting a competition of lysozyme and IgG with the Ca^2+^/CaM-stimulus that induces CaMKII/GluN2B binding. However, lysozyme negatively regulated binding even when it was instead induced by CaMKII T286 phosphorylation. Alternative modes of competition would be with CaMKII or GluN2B, and the negative effects of lysozyme and IgG indeed also correlated with specific or non-specific binding to the immobilized GluN2B. Thus, the effect of any specific crowding reagent can differ, depending on its additional direct effects on CaMKII/GluN2B binding. However, the results of this study also indicate that, in principle, macromolecular crowding enhances CaMKII binding to GluN2B.

## Introduction

CaMKII is a ubiquitous mediator of cellular Ca^2+^-signals and is best known for its functions in the regulation of synaptic plasticity underlying learning, memory and cognition (for review see [1], [2]). Long-term potentiation (LTP) of synaptic strength requires the CaMKIIα isoform [3] and its Ca^2+^-independent “autonomous” activity that is generated by autophosphorylation at T286 [4], [5]. Additionally, normal physiological LTP requires CaMKII binding to the NMDAR subunit GluN2B [6], [7], specifically to a site located around S1303 of the cytoplasmic C-tail of GluN2B [8]. This CaMKII binding to GluN2B can be induced either by Ca^2+^/CaM-stimulation [9]–[11] or by CaMKII T286 autophosphorylation [8], [9], [11], and is also positively regulated by occupation of the CaMKII nucleotide binding pocket [12]–[14]. Once CaMKII is bound to GluN2B, it remains partially “autonomous”, i.e. partially active even after removal of Ca^2+^/CaM, and this effect is independent of T286 phosphorylation [9], [10]. This regulation can be explained by the GluN2B binding-site on CaMKII, termed the T-site [9], [10]. In the basal, inactive state of CaMKII, this T-site is blocked by the autoregulatory domain, specifically by the region around T286 [15], [16]. Binding of Ca^2+^/CaM displaces the autoregulatory region, which makes the T-site accessible for GluN2B binding and makes T286 accessible for autophosphorylation. Then, either occupation of the T-site or T286 phosphorylation prevents complete re-association of the autoregulatory domain after the Ca^2+^/CaM-stimulus has subsided, thereby supporting continued “autonomous” CaMKII activity (for review see [1]). However, “autonomous” CaMKII is by no means fully active: T286-phosphorylated CaMKII can still be significantly further stimulated by Ca^2+^/CaM (∼5-fold; [17], [18]), and the partial autonomous activity of GluN2B-bound CaMKII is likely even lower, due to partial interference with substrate binding [9], [14] (for review see [1]). Thus, compared to regulation of kinase activity, a more important function of the CaMKII/GluN2B interaction in LTP may be the targeting of CaMKII to synaptic NMDARs (for review see [1], [2]).

Negative regulation of the CaMKII/GluN2B interaction can be caused by two distinct CaMKII-mediated phosphorylation reactions. First, CaMKII can phosphorylate GluN2B at S1303 [19], which in turn inhibits CaMKII binding to GluN2B [8], [11], [12]. For this reason, CaMKII binding to GluN2B is enhanced less by the presence of ATP compared to ADP [12], as ADP cannot be used for the inhibitory phosphorylation reactions. However, due to the positive effect of direct nucleotide binding to CaMKII, ATP still enhances the binding to GluN2B compared to conditions without any nucleotide present [12], [13]. Second, CaMKII autophosphorylation at T305/306 prevents binding of Ca^2+^/CaM to CaMKII [20], [21], and can thereby also inhibit CaMKII binding to GluN2B [12], as Ca^2+^/CaM is required to induced binding to GluN2B, either directly or via autophosphorylation at T286. Notably, CaMKII that is phosphorylated first at T286 and at then at T305/306 still binds to GluN2B [22]. However, T305/T306 phosphorylation reduces phospho-T286-induced CaMKII binding to GluN2B also independently from any effect on Ca^2+^/CaM, (Coultrap and Bayer, unpublished observation).

Notably, the extensive biochemical studies on the regulation of the CaMKII/GluN2B interaction described above have not included conditions that mimic the macromolecular crowding found within cells. In principle, such molecular crowding is expected to enhance binding reactions (for review see [23]–[25]). However, this is based on the assumption that the molecular crowding reagent is inert to the binding reaction, which needs to be tested for any specific crowding reagent. For CaMKII, there is an additional complication to a straightforward prediction. CaMKII forms 12meric holoenzymes, with the C-terminal association domains forming a central hub and the N-terminal kinase domains radiating outward (for review see [1]). Importantly, while CaMKII holoenzymes are in an extended open conformation in typical biochemical dilutions, molecular crowding conditions favor a compact closed conformation in which the kinase domains fold back to interact with the association domains, a conformation that hinders Ca^2+^/CaM binding [15].

Here, we tested the effect of several molecular crowding reagents on CaMKII binding to GluN2B *in vitro*. While most crowding reagents had a positive effect (BSA, dextran-10, dextran-70), two had a negative effect (lysozyme, IgG) and one had no statistically significant effect (PVP-40). Importantly, while nucleotide and macromolecular crowding (with BSA or dextran) both significantly enhanced Ca^2+^/CaM-stimulated binding to GluN2B, neither was sufficient to induce binding in the absence of Ca^2+^/CaM or T286 phosphorylation. Thus, the constitutive presence of nucleotide and molecular crowding within cells can affect binding, but does not alleviate the strict dependence of CaMKII/GluN2B interaction on Ca^2+^-mediated signaling.

## Materials and Methods

### Materials

CaM, GST-GluN2B-C and CaMKIIα were purified after bacterial or baculovirus/Sf9 cell expression, as described in detail elsewhere [9], [26], [27]. Pipes, Tween-20, CaCl_2_, MgCl_2_, EGTA, ADP, Ponceau S, dextran-10 and −70, PVP-40, non-specific rabbit IgG (catalog number: I5006, lot SLBD3695V), chicken lysozyme (catalog number: L7651, lot SL07134), and BSA (catalog number: A2153, lot SLBC8307) were obtained from Sigma. NaCl and anti-GST coated microtiter plates were obtained from Thermo-Fisher. Staurosporine was purchased from LC labs. ATP was purchased from Calbiochem.

### Statistical analysis

All quantified data are shown as mean ± standard error of the mean. Statistical analysis was performed in SPSS 22 (IBM). All data were subjected to a Shapiro-Wilk test for normality and Leven's test of equal variance. Data meeting parametric criteria were analyzed using a one-way ANOVA for three or more groups followed by Tukey's honest significant difference test for multiple comparisons. An un-paired two-tailed student's t-test was used for comparisons between two groups. For non-parametric data, an Independent Samples Kruskal-Wallis test followed by a Dunn-Bonferroni test for multiple comparisons was employed. An un-paired two-tailed student's t-test corrected for non-equal variance was used for comparisons between two non-parametric groups. Alpha was set to p<0.05 to determine significance. Experimental outliers were identified in SPSS (greater than 2 standard deviations from the group mean) and eliminated from all analysis.

### CaMKII binding to GluN2B immobilized in microtiter plates

CaMKII binding to GST-GluN2B-C (containing the GluN2B cytoplasmic C-terminus, amino acids 1120-1482) immuno-immobilized in anti-GST-coated microtiter plate wells was done as described previously [9], [12], [13], [27] with some modifications. Plates were washed four times with PST (50 mM PIPES pH 7.12, 150 mM NaCl, 0.05% Tween-20) prior to GST-GluN2B binding. GST-GluN2B (300 nM; an over-saturating concentration) was incubated in wells in a 50 µl PST solution containing 0.1% BSA for one hour under gentle agitation at room temperature. Plates were then washed four more times and blocked with 100 µl 5% BSA in PST for one hour under gentle agitation at room temperature. After removal of blocking solution, plates were washed once more with PST. Then, molecular crowding reagents (Lysozyme, BSA, dextran-10 and −70, PVP-40, or IgG; 30 µl) were added, followed by binding buffer (10 µl) and CaMKII (10 µl), for a final concentration of 40 nM CaMKIIα subunits, 100 mg/ml crowding reagent, 50 mM PIPES pH 7.12, 150 mM NaCl, 0.1% Tween-20, 1 mM CaCl_2_, 1 µM CaM, 9.5 mM MgCl_2_ and 100 µM ADP. This binding reaction was incubated for 15 min under gentle agitation at room temperature. Unbound CaMKIIα was discarded and plates were washed in PST containing 1 mM EGTA four to eight times to remove remnants of high concentration molecular crowding agents. 60 µl of loading buffer containing 2% SDS was then added to the wells and samples were incubated at 95°C for 10 minutes to dissociate bound proteins from the plates. Samples were then analyzed by SDS-PAGE and immuno-blotting.

### CaMKII binding to GluN2B in glutathione-sepharose pull-down assays

Glutathione coated magnetic beads (Pierce #88821) were washed three times in ice-cold phosphate buffered saline (PBS), diluted to a 50% slurry in PBS, and added to 0.5 ml tubes at a final bed volume of 1 µl. GST-GluN2B (1 µM) in a 50 µl PST solution containing 0.1% BSA was incubated with the beads for one hour under gentle agitation at room temperature. Beads were fully re-suspended periodically during the binding reaction. Beads were next separated by magnetic interaction and washed three times with PST. Beads were then blocked in 50 µl 5% BSA in PST for 30 minutes under gentle agitation at room temperature. After removal of the blocking solution, plates were washed once more with 100 µl PST. Then, molecular crowding reagents (Lysozyme, BSA, dextran-70; 30 µl) were added, followed by binding buffer (10 µl) and CaMKII (10 µl), for a final concentration of 40 nM CaMKIIα subunits, 100 mg/ml crowding reagent, 50 mM PIPES pH 7.12, 150 mM NaCl, 0.1% Tween-20, 1 mM CaCl_2_, 1 µM CaM, 9.5 mM MgCl_2_ and 100 µM ADP. (Same as plate binding experiments.) This binding reaction was incubated for 15 min under gentle agitation at room temperature, and beads were fully re-suspended twice at 5 and 10 minutes. Unbound CaMKII was discarded and beads were separated and washed in PST containing 1 mM EGTA eight times to remove remnants of high concentration molecular crowding agents. 40 µl loading buffer containing 2% SDS was then added to the beads and samples were incubated at 95°C for 10 minutes to dissociate bound proteins. Samples were then analyzed by SDS-PAGE and immuno-blotting.

### SDS-PAGE and immuno-detection

Gel electrophoresis was performed using 10 or 15% polyacrylamide gels and transferred to PVDF membranes, as previously described [27], [28]. Immuno-detection was then preformed using CBα2 anti-CaMKIIα 1∶5000 (available at Invitrogen, but made in house), anti-pT286 CaMKII 1∶2000 (PhosphoSolutions), or anti-GST 1∶5000 (Millipore) followed by Amersham ECL anti-mouse IgG, horseradish peroxidase-linked secondary 1∶5000 (GE Healthcare) or goat anti-rabbit IgG horseradish peroxidase conjugate 1∶2000 (Bio-Rad). The dilution buffer was TBS-T (20 mM Tris HCl pH 7.4, 150 mM NaCl and 0.1% Tween-20). Blots using CBα2 and anti-pT286 were blocked in 5% milk in TBS-T with antibody and secondary dilutions in 1% milk in TBS-T. Anti-GST blots were blocked in 5% BSA in TBS-T and antibody and secondary dilutions were in 1% BSA in TBS-T. Blots were developed using chemi-luminescence (Super Signal West Femto, Thermo-Fisher) and imaged using the ChemiImager 4400 system (Alpha-Innotech). Densitometry was calculated in FIJI (NIH). Two or more control conditions were loaded per gel, and the relative immuno-detection value (IDV) was normalized as a percent of the average of all control conditions for the same blot, which was set at a value of one to allow comparison between multiple experiments.

### CaMKII autophosphorylation reaction and binding

CaMKIIα autophosphorylation at T286 was induced for ten minutes on ice, as previously described [5], [18], [27], in 50 mM PIPES pH 7.12, 0.1% BSA, 10 mM MgCl_2_, 100 µM ATP, 1 mM CaCl_2_, and 2 µM CaM. Then, CaMKII activity was inhibited with 10 µM staurosporine and Ca^2+^ was chelated with 2 mM EGTA (final concentrations after a dilution with two volumes of 50 mM PIPES pH 7.12, 0.1% BSA). This protocol has been shown to selectively phosphorylate T286, with minimal or no phosphorylation at other sites [5]. Then, binding of phopho-T286 CaMKII to GluN2B was done as described above, but in the presence of 1.5 mM EGTA, 2 µM staurosporine, and without addition of Ca^2+^/CaM or nucleotide.

### Protein staining and CaM overlays

For silver staining experiments, gels were fixed and stained following the manufacturer protocol (Silver Stain Plus, BioRad, catalog number 161-0449). Membranes were stained for total protein using Ponceau S (0.1% in 1% acetic acid) for five minutes, then washed with water prior to imaging. Images were acquired using the ChemiImager 4400 system (Alpha-Innotech). CaM overlays were done as previously described [29] with minor modifications. Membranes were incubated with biotinylated CaM (CalBiochem), diluted 1∶2400, for 30 min at room temperature in TBS-T, with or without 1 mM CaCl_2_. Membranes were then washed with TBS-T, incubated in VectaStain (Vector Laboratories) for 30 minutes, and again washed in TBS-T (at each step with or without 1 mM CaCl_2_, matching the initial incubation condition). Blots were developed using chemi-luminescence as described for immuno-detection, but again with the addition of 1 mM CaCl_2_.

## Results

### Molecular crowding with lysozyme and BSA has opposite effects on CaMKII binding to GluN2B

**Figure 1:**
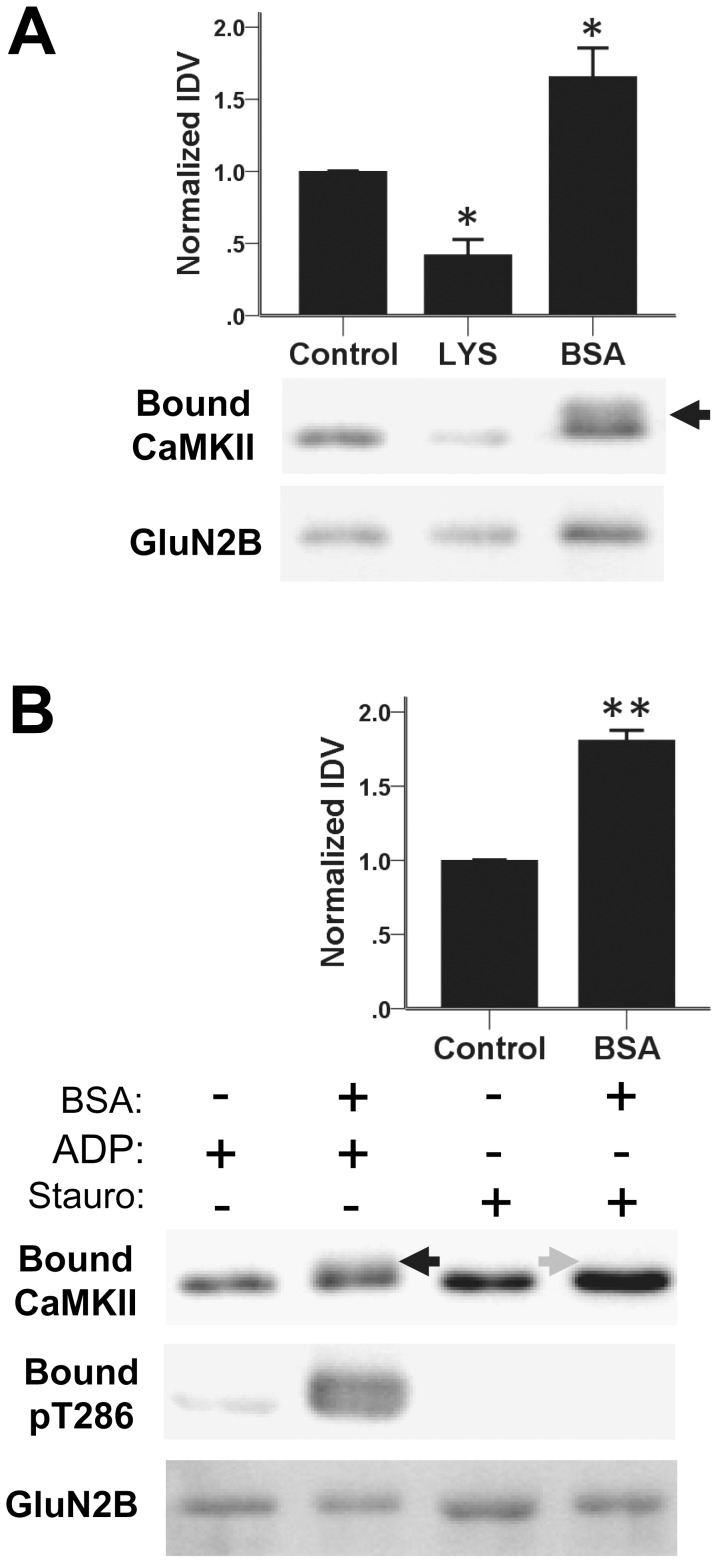
Differential effects of BSA and lysozyme molecular crowding agents in CaMKII to GluN2B binding. *A*, CaMKIIα (40 nM subunits) binding to GST-GluN2B-C that was immuno-immobilized on anti-GST coated microtiter wells was induced by Ca^2+^/CaM (1 mM/1 µM) in the presence of ADP (100 µM) for 15 min at room temperature. Bound CaMKII was eluted and detected by Western-analysis, and quantified by normalized immuno-detection values (IDV). Macromolecular crowding with lysozyme (100 mg/ml) decreased CaMKII binding to GluN2B, while BSA (100 mg/ml) instead increased binding. n = 4; *: p<0.05 in one-way ANOVA followed by Tukey's HSD. Bar graphs indicate mean ± s.e.m, and GST-GluN2B detection is shown as a loading control. Black arrow indicates a band shift that suggested phosphorylation of CaMKII. *B*, Phosphorylation of CaMKII in the presence of BSA molecular crowding was verified using a phospho-T286-specific antibody (indicating presence of ATP), and was inhibited by addition of staurosporine (Stauro). Black arrow represents band shift corresponding to anti-phospho-T286 reactivity. Grey arrow indicates loss of band shift in the presence of staurosporine, verified by lack of immuno-reactivity to anti-phospho-T286. Anti-phospho-T286 blots are from the same experiment, but run on a separate gel to avoid reprobing blots at the same molecular weight. Inhibiting autophosphorylation of T286 did not prevent the positive effects of BSA molecular crowding on CaMKII to GluN2B binding. n = 3; **: p<0.01 in two-tailed students t-test. Bar graphs indicate mean ± s.e.m, and GST-GluN2B detection is shown as a loading control.

The effect of molecular crowding on CaMKII binding to GluN2B was here first tested using lysozyme and BSA, each at 100 mg/ml. As molecular crowding depends not only on the number but also the size of the crowding agent molecules, using equal weight per volume of the crowding agent was considered to provide a better comparison than using equal molarities. GST-fusion protein with the cytoplasmic C-terminus of GluN2B (GluN2B-C; amino acids 1120–1482) was immobilized on anti-GST-coated microtiter plate wells, and binding of CaMKII (40 nM subunits) was induced by Ca^2+^/CaM (1 mM/1 µM) in the presence of ADP (100 µM) for 15 min at room temperature. After extensive washes, bound CaMKII was eluted and then detected by Western-blot and quantified (Fig. 1A). Surprisingly, lysozyme and BSA had opposite effects on CaMKII binding to GluN2B. While lysozyme reduced the binding, BSA instead enhanced it (Fig. 1A) [F(2,11) = 22.50, p<0.001; difference from control: lysozyme, p = 0.03, BSA, p = 0.02]. A dose response indicated that both the positive effect of BSA and the negative effect of lysozyme increased with increasing concentrations (Fig. S1).

Notably, the CaMKII that bound in the presence of BSA showed a band-shift indicative of autophosphorylation (Fig. 1A). This suggested that the BSA preparation contained ATP, which would allow T286 autophosphorylation in the presence of the Ca^2+^/CaM stimulus that was added to induce the binding to GluN2B. Indeed, after binding in the presence of BSA, the bound CaMKII was significantly autophosphorylated at T286, as detected by Western-analysis with a phospho-T286-specific antibody (Fig. 1B).

In order to test if T286 phosphorylation accounted for the positive effect of BSA on CaMKII binding to GluN2B, staurosporine was used to inhibit phosphorylation during the binding reaction (again in the presence of Ca^2+^/CaM). Staurosporine inhibits CaMKII activity in a nucleotide-competitive manner, but mimics rather than inhibits the positive-regulatory effect of nucleotide on CaMKII binding to GluN2B (making addition of nucleotide to stauroporine-containing binding reactions unnecessary) [13]. While staurosporine completely blocked T286 phosphorylation, it did not prevent the significant enhancing effect of BSA on the CaMKII/GluN2B interaction [t(2) = 12.27, p = 0.007] (Fig. 1B). Thus, T286 phosphorylation does not explain the opposite effects of molecular crowding by lysozyme versus BSA.

### Two differently sized dextrans both enhance CaMKII/GluN2B binding

**Figure 2:**
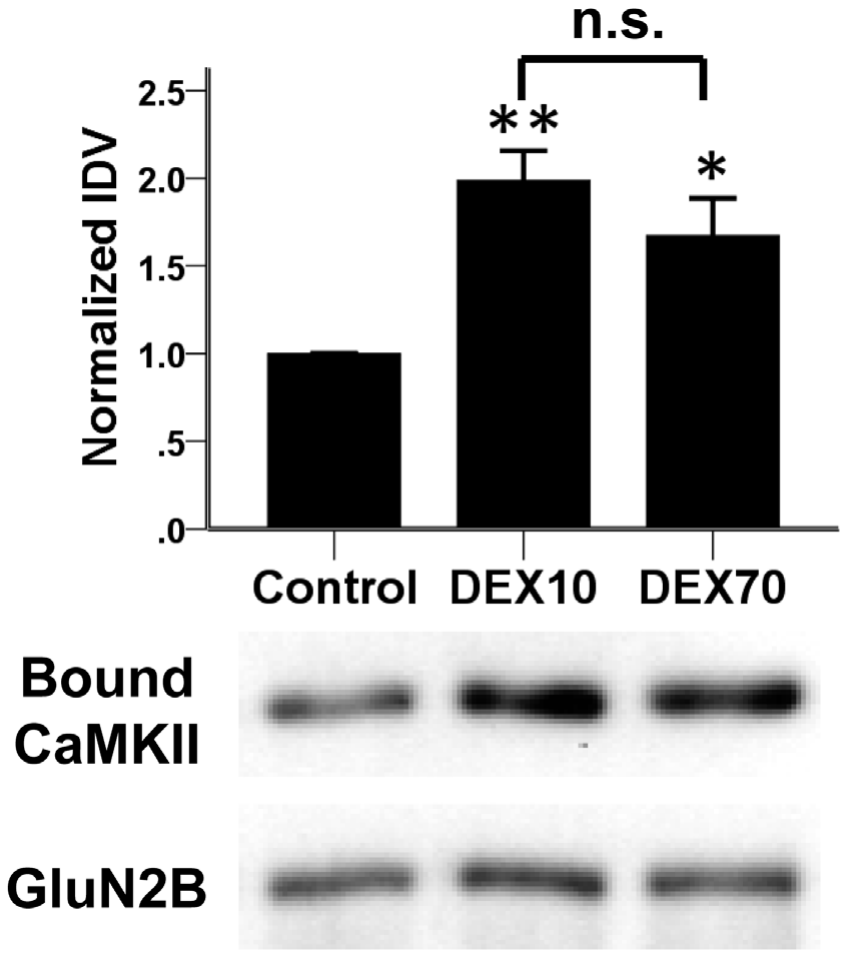
Two differently sized dextrans both enhance CaMKII binding to GluN2B, indicating that the differential effects of BSA and lysozyme were not due to size differences. Ca^2+^/CaM-stimulated CaMKII binding to GluN2B was tested as in Figure 1A. Molecular crowding with dextran-10 (DEX10) or dextran-70 (DEX 70), both at 100 mg/ml, both increased CaMKII binding to GluN2B to an equal extent. n = 6–7; *: p<0.05, **: p<0.01 in one-way ANOVA followed by Tukey's HSD; n.s.: no significant difference between the two dextrans. Bar graphs indicate mean ± s.e.m, and GST-GluN2B detection is shown as a loading control.

One difference between lysozyme (14 kDa) and BSA (66 kDa) as molecular crowding reagents is their size. In order to test if the size of a crowding reagent can determine its effect on CaMKII binding to GluN2B, two differently sized dextrans were compared. Dextran-10 and dextran-70 were chosen, as their molecular weight corresponds approximately to that of lysozyme and BSA, respectively. At 100 mg/ml, both dextrans significantly enhanced the Ca^2+^/CaM-stimulated CaMKII binding to GluN2B to equal extents our assay (Fig. 2) [F(2,19) = 10.44, p = 0.001; difference from control: dextran-10, p = 0.001; dextran-70, p = 0.016; no difference between the dextrans]. Thus, size of a crowding reagent is not a direct predictor of its effect on the CaMKII/GluN2B interaction.

### Ca^2+^/CaM binds to lysozyme but not BSA

**Figure 3:**
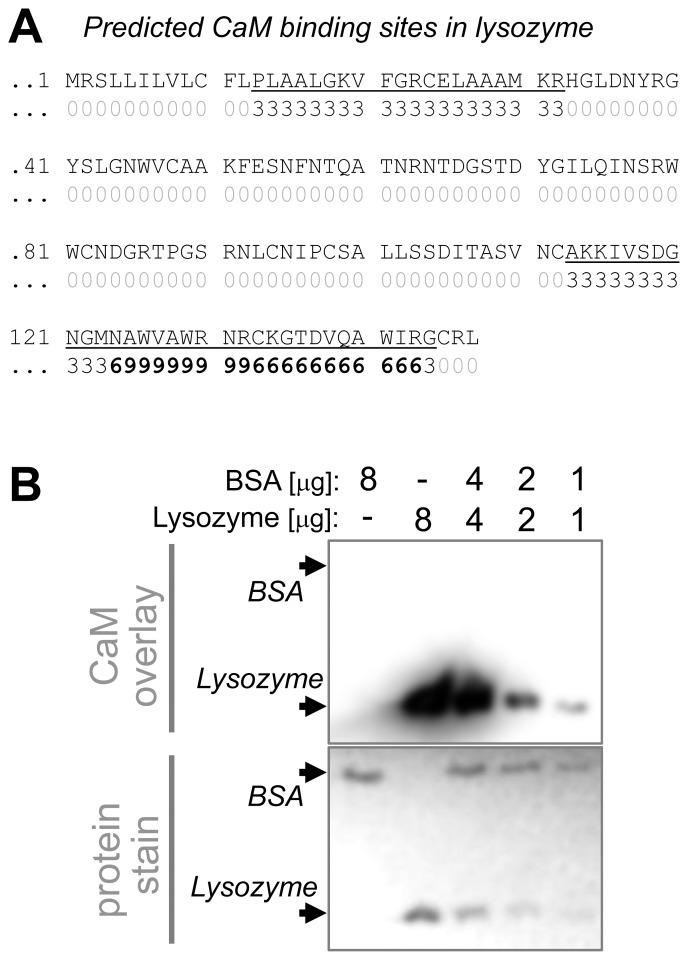
Lysozyme but not BSA binds Ca^2+^/CaM. *A*, The chicken lysozyme amino acid sequence with predicted CaM-binding sites marked. Prediction utilized the “Calmodulin Target Database” (http://calcium.uhnres.utoronto.ca/ctdb/ctdb/home.html). Numbers from 1 to 9 indicate increasing likelihood of predicted CaM binding. *B*, Lysozyme and BSA (0, 1, 2, 4, and 8 µg) were subjected to SDS-PAGE and transferred to a PVDF membrane. The membrane was first stained for total protein (ponceau, bottom panel), then incubated with biotin-labeled CaM in presence of CaCl_2_. Bound CaM was detected by chemi-luminescence (top panel). Ca^2+^/CaM bound Lysozyme, but not BSA.

Sequence analysis (using the “Calmodulin Target Database”; http://calcium.uhnres.utoronto.ca/ctdb/ctdb/home.html) of chicken lysozyme revealed two putative CaM binding sites, one of them with the highest possible likelihood indicator (Fig. 3A). Lysozyme binding to Ca^2+^/CaM could create competition for the stimulus that induces CaMKII binding to GluN2B. However, sequence analysis also yielded two potential sites with similar likelihood indicators for BSA (not shown). Thus, we tested binding of biotinylated Ca^2+^/CaM to both lysozyme and BSA in a blot overlay assay (Fig. 3B). Clear specific Ca^2+^/CaM binding was detected for lysozyme, but not BSA. Even exposures that overexposed the binding signal for lysozyme did not show any signal for BSA (Fig. 3B). Thus, lysozyme but not BSA can compete with CaMKII for binding to Ca^2+^/CaM, the stimulus used to induce CaMKII binding to GluN2B.

Compared to CaMKII, the CaM binding detected for lysozyme was much lower (∼500-fold; Fig. S2). However, the lysozyme concentration in the GluN2B binding assays used in this study was 50,000-fold higher compared to the CaMKII concentration. Thus, the assay conditions used here would allow for effective competition of lysozyme with CaMKII for Ca^2+^/CaM binding, despite the relatively low Ca^2+^/CaM- affinity of lysozyme.

### Lysozyme also inhibits phospho-T286-induced CaMKII/GluN2B binding

**Figure 4:**
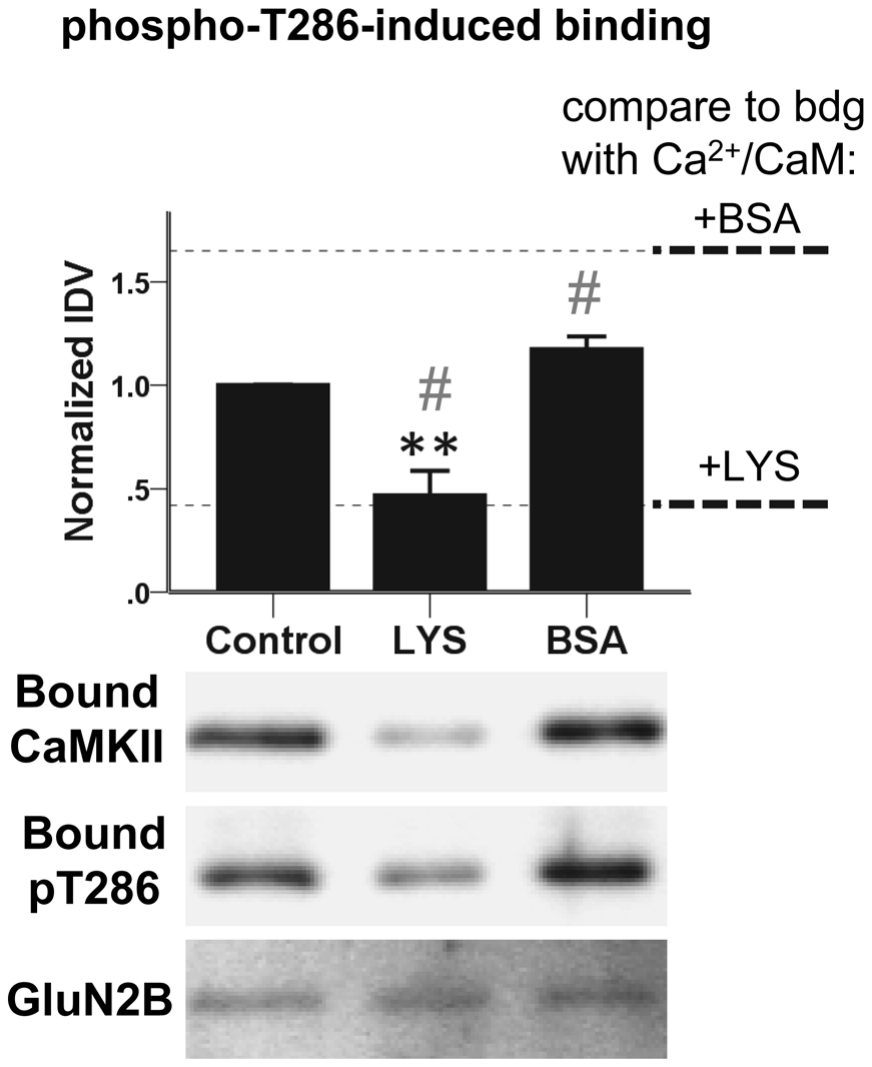
Induction of binding by T286 phosphorylation, instead of Ca^2+^/CaM. CaMKIIα was pre-incubated with ATP and Ca^2+^/CaM to induce autophosphorylation at T286; then, Ca^2+^ was chelated with EGTA and further phosphorylation was inhibited by staurosporine. Phospho-T286 CaMKIIα (40 nM subunits) was bound to GST-GluN2B as in Figure 1A, but in the presence of 1.5 mM EGTA, 2 µM staurosporine, and without addition of Ca^2+^/CaM or nucleotide. Lysozyme (100 mg/ml) decreased CaMKII binding to GluN2B. T286-phosphorylation was verified using a phospho-T286-specific antibody. Phospho-T286 immunoblots are from the same experiment run on a separate gel to avoid reprobing blots at the same molecular weight. Dotted lines represent IDVs from CaMKII/GluN2B binding when instead stimulated with Ca^2+^/CaM and nucleotide (from Figure 1A). n = 6 or 7 per group; **: p<0.01, difference from control, one-way ANOVA followed by Tukey's HSD. #: no difference compared to the same binding condition, but with binding induced by Ca^2+^/CaM (compare Fig. 1A). Bar graphs indicate mean ± s.e.m, and GST-GluN2B detection is shown as a loading control.

If molecular crowding enhances CaMKII binding to GluN2B in principle, with the negative effect of lysozyme due to competition with CaMKII for Ca^2+^/CaM binding, lysozyme should no longer be inhibitory when CaMKII/GluN2B interaction is induced by T286 phosphorylation instead of Ca^2+^/CaM. Thus, for additional binding reactions, CaMKII was autophosphorylated at T286, and Ca^2+^ was chelated with EGTA before the binding. Removal of Ca^2+^/CaM from T286-phosphorylated “autonomous” CaMKII induces secondary autophosphorylation at other residues, including T305/306 [5], [20], [21]. As such secondary phosphorylation may interfere with binding to GluN2B [22] and results in additional band-shifts that cause CaMKII to run in a multi-band pattern [5], [21], secondary phosphorylation at additional residues was blocked by addition of staurosporine during the Ca^2+^ chelation and the subsequent binding reaction. Indeed, this treatment caused the T286-phosphorylated GluN2B-bound CaMKII to run as a single band (Fig. 4). Although the enhancement effect of BSA in the phospho-T286-induced CaMKII binding failed to reach significance in the ANOVA model, it was significant by t-test, [t(5) = −2.2813, p = 0.04] (Fig. 4). More importantly, there was no difference between the BSA effects on binding induced by phospho-T286 compared to induction by Ca^2+^/CaM [t(3.59) = −2.32, p = 0.09]. However, contrary to our expectation, molecular crowding with lysozyme significantly reduced CaMKII binding to GluN2B even when the binding was induced by T286 phosphorylation instead of Ca^2+^/CaM [F(2,18) = 19.983, p<0.001; difference from control: lysozyme, p = 0.001, no difference from binding induced by Ca^2+^/CaM] (Fig. 4). Thus, lysozyme can negatively impact the CaMKII/GluN2B interaction independent from competition for Ca^2+^/CaM.

### Non-specific binding of lysozyme in the plate assay

**Figure 5:**
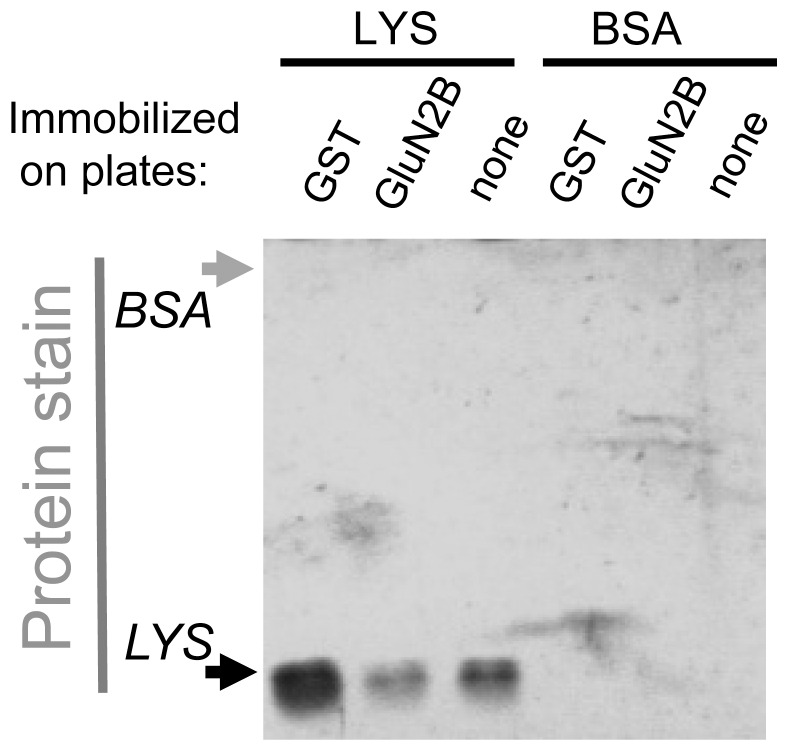
Lysozyme but not BSA binds to the GST immobilization scaffold. 100 mg/ml Lysozyme (6.8 mM) or BSA (1.5 mM) were tested for binding to immuno-immobilized GST-GluN2B-C under the same conditions as in Figure 1A. Samples were eluted and subjected to SDS-PAGE, after which the gel was fixed and stained for total protein (silver stain). Binding was detected for lysozyme but not BSA. Lysozyme binding was detected also to immobilized GST (without GluN2B fusion) and to empty wells, indicating non-specific binding to the immobilization scaffold.

An alternative explanation for the negative effect of lysozyme on CaMKII/GluN2B binding would be direct binding of lysozyme to either CaMKII or GluN2B, which could cause another mode of competition. Due to the high concentration of molecular crowding reagents, even a low-affinity binding could be sufficient for such competition. As low-affinity binding could be reversed during the washes required in our binding assay, it may not be readily detectable. Nevertheless, we decided to test if such binding is detectable. As expected, no binding of BSA to immobilized GST-GluN2B was detected (Fig. 5). By contrast, binding of lysozyme was readily detectable by silver stain (Fig. 5). However, this binding was not GluN2B-specific, as lysozyme binding was also detected in control wells with GST only or without any immobilized protein (Fig. 5).

In order to test if the non-specific binding of lysozyme to the microtiter plate wells caused the negative effect of lysozyme on CaMKII/GluN2B binding, we decided to utilize another binding assays, pull-down of GST-GluN2B with glutathione-sepharose beads. In this assays system, molecular crowding with dextran-70 enhanced CaMKII/GluN2B binding (Fig. S3A), as expected from the results obtained with the plate binding assays (see Fig. 2). However, lysozyme still showed both a negative effect on CaMKII/GluN2B binding (Fig. S3A) and a non-specific binding to the scaffold of the assay (Fig. S3B); thus, both findings remain correlated also in the glutathione-sepharose pull-down assay.

Somewhat surprisingly, and in contrast to the plate binding assay, the pull-down assay detected BSA binding to GST-GluN2B (Fig. S3B). No BSA binding to GST or glutathione-sepharose alone was detected (Fig, S3B), indicating specific binding to GluN2B. Compared to the plate binding assays, the pull-down assays contained 3-fold the amount of GluN2B, increasing the ratio of GluN2B to CaMKII (the latter was kept constant between the two assays), possibly allowing enhanced binding of BSA (and better detection of such binding). Consistent with the expectation that such BSA binding to the immobilized GluN2B could interfere with CaMKII/GluN2B binding, the enhancing effect of BSA seen in the plate assays was lost in the pull-down assays (Fig. S3C).

### Differential effects of IgG and PVP-40

**Figure 6:**
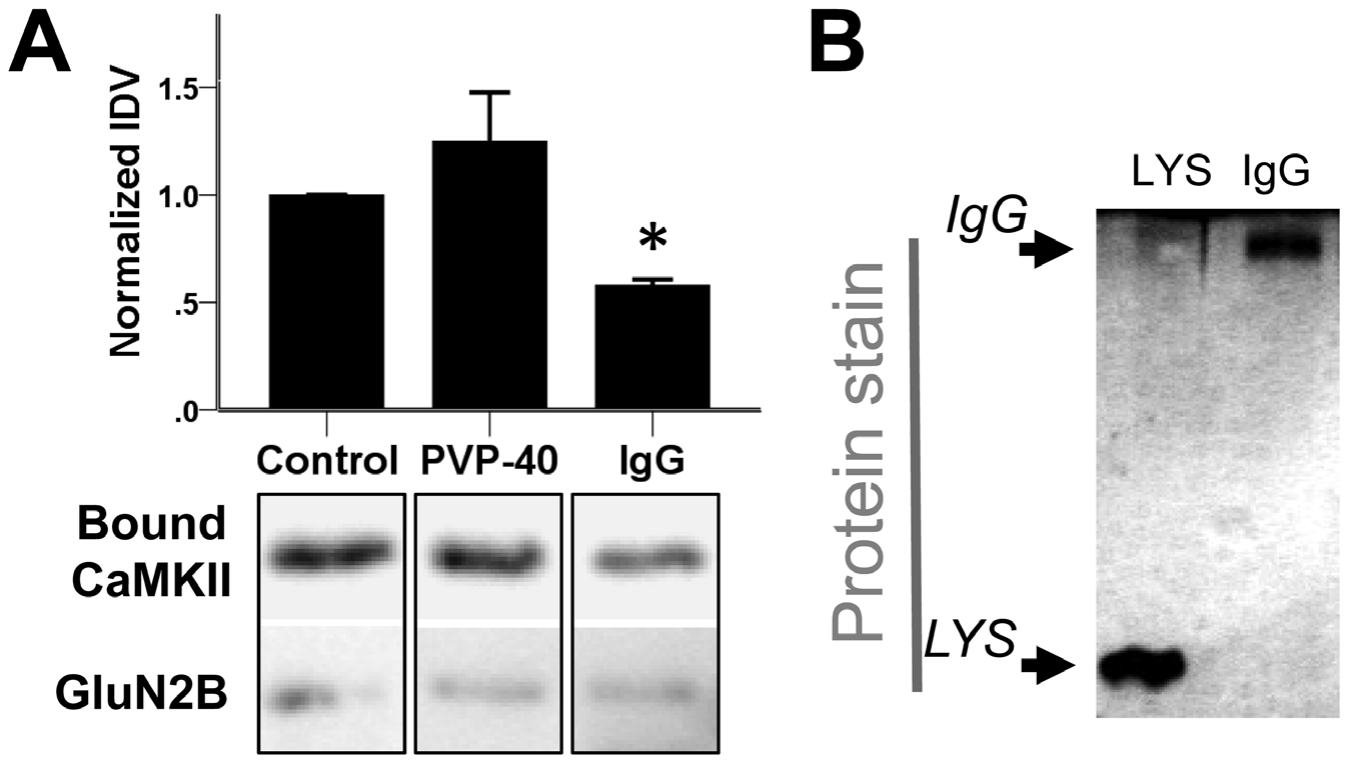
IgG binds to GluN2B and decreases CaMKII binding to GluN2B. *A*, Ca^2+^/CaM-stimulated CaMKII binding to GluN2B was tested as in Figure 1A. Molecular crowding with PVP-40 (100 mg/ml) had no effect on CaMKII binding to GluN2B, while rabbit immunoglobulin (IgG) (100 mg/ml) decreased binding. n = 7 (PVP-40) or 4 (IgG); *: p<0.05 in Kruskal Wallis test followed by Dunn-Bonferroni multiple comparison. Bar graphs indicate mean ± s.e.m, and GST-GluN2B detection is shown as a loading control. The immuno-detection examples are cropped from the same exposure of the same blots. *B*, 100 mg/ml IgG or Lysozyme were tested for binding to immuno-immobilized GST-GluN2B-C under the same conditions as in Figure 1A. Samples were eluted and subjected to SDS-PAGE, after which the gel was fixed and stained for total protein (silver stain). Binding was detected for both IgG and lysozyme.

With three molecular crowding reagents enhancing CaMKII binding to GluN2B and one reducing it, we decided to test two more reagents, the polymer PVP-40 and the protein rabbit IgG, again at 100 mg/ml. While PVP-40 appeared to enhance binding, this effect was not statistically significant. By contrast, IgG reduced CaMKII binding to GluN2B (Fig. 6A) [H(2,16) = 9.17, p = 0.014; difference from control: IgG, p = 0.01].

Thus, we tested if the negative effect of IgG on CaMKII binding to GluN2B could be explained by direct binding of IgG to the immobilized GluN2B, similar to what was seen for lysozyme. Indeed, bound IgG heavy chain was clearly detected by silver stain (Fig. 6B).

Additionally, we decided to test for binding of biotinylated CaM to IgG in a blot overlay assay. In the presence of Ca^2+^, a CaM binding signal was detected for the IgG light chain, but not for the IgG heavy chain or BSA (Fig. S4). However, compared to lysozyme, this binding signal was rather weak (Fig. S4). Without the addition of Ca^2+^, at least a weak CaM binding signal was seen for all proteins tested (Fig. S4). While lysozyme showed the strongest CaM binding signal of the crowding agents also in the absence of Ca^2+^, it was the only tested protein for which the binding signal was noticeably higher in the presence of Ca^2+^.

Together, these results indicate that most but not all molecular crowding reagents enhance CaMKII binding to GluN2B. The exceptions, lysozyme and IgG, were correlated with their specific or non-specific binding to Ca^2+^/CaM and to immobilized GST-GluN2B. The latter binding provides a more likely explanation for the negative effects in both cases: Lysozyme decreased CaMKII binding to GluN2B even when binding was stimulated by T286 phosphorylation instead of Ca^2+^/CaM, and IgG showed only very weak Ca^2+^/CaM-binding, even when compared to lysozyme.

### Nucleotide and molecular crowding agents do not alleviate the stimulation requirement of CaMKII/GluN2B binding

**Figure 7:**
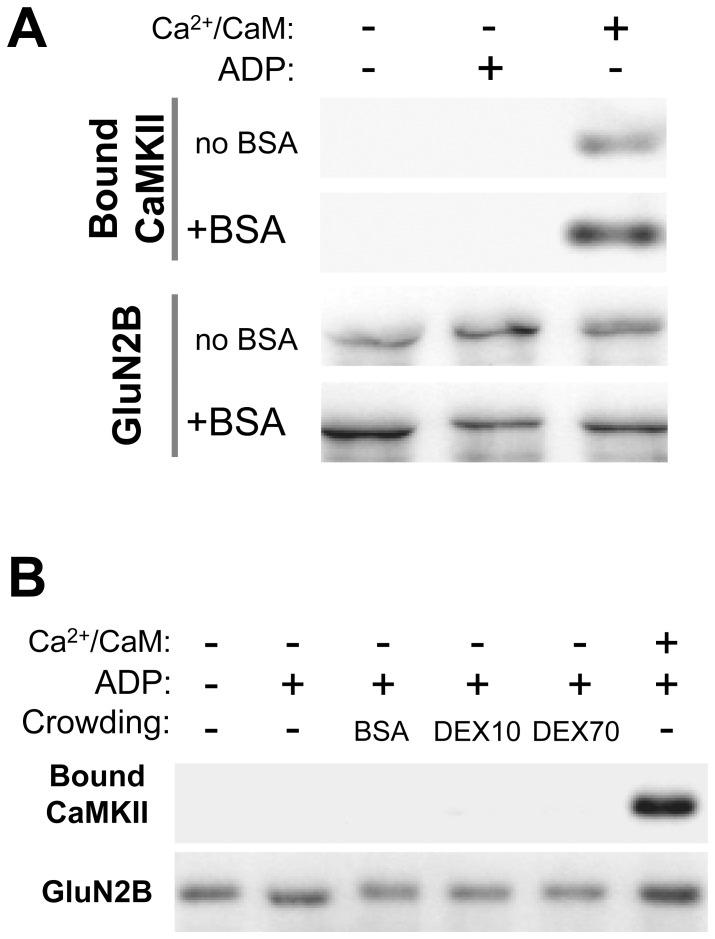
Ca^2+^/CaM stimulation is still required for CaMKII to GluN2B binding. *A*, CaMKIIα (40 nM subunits) was incubated with immuno-immobilized GST-GluN2B-C as in Figure 1A, but in the presence or absence of Ca^2+^/CaM (1 mM/1 µM), ADP (100 µM), or BSA (100 mg/ml). Bound CaMKII was eluted and detected by Western-analysis. BSA and ADP alone were insufficient to induce CaMKII to GluN2B binding. GST-GluN2B detection is shown as a loading control. Representative images are from the same experiment and Western-blots. *B*, CaMKIIα (40 nM subunits) was incubated with immuno-immobilized GST-GluN2B-C as in panel A. Different crowding agents, BSA, dextran-10 (DEX10) and dextran-70 (DEX70) (all at 100 mg/ml) in the presence of ADP (100 µM) were not sufficient to induce detectable CaMKII binding to GluN2B without CaMKII stimulation.

Macromolecular crowding with BSA or with dextran-10 or −70 significantly enhanced CaMKII binding to GluN2B. Previous studies showed that CaMKII/GluN2B binding is also enhanced by nucleotides, with the strongest effect elicited by ADP [12]. As molecular crowding and high nucleotide concentrations are continuous basal conditions within cells, an important question was if these conditions are sufficient to induce CaMKII binding to GluN2B even in absence of CaMKII stimulation, a situation that would effectively uncouple induction of this binding from cellular Ca^2+^-signaling. In the absence of stimulation by Ca^2+^/CaM, neither BSA nor ADP nor a combination of both was sufficient to induce any detectable CaMKII binding to GluN2B in our assay (Fig. 7A). In addition, there was no detectable binding in the presence of ADP and either dextran-10 or dextran-70 (Fig. 7B). Thus, the positive regulation of CaMKII binding to GluN2B provided by several molecular crowding agents and by nucleotide does not alleviate the strict requirement for CaMKII stimulation in order to induce the CaMKII/GluN2B interaction.

## Discussion

The regulated binding of CaMKII to the NMDAR subunit GluN2B is thought to mediate stimulus-induced targeting of CaMKII to synapses, and is functionally important for enhancement of synaptic strength (for review see [1], [2]). The results of this study indicate that mimicking the macromolecular crowding conditions found within cells in principle enhances CaMKII binding to GluN2B, consistent with the expectation that such crowding favors the interaction between binding partners (for review see [23]–[25]). However, some crowding reagents, lysozyme and IgG, instead even decreased binding. Initially, this effect appeared to be explained by an unexpected Ca^2+^/CaM binding to lysozyme (and to a lesser extent to IgG), which could compete for the stimulus that induced the binding. However, lysozyme had a negative effect even when binding was induced instead by CaMKII autophosphorylation at T286, without any free Ca^2+^ present in the binding reaction. Thus, the effect of other specific molecular crowding reagents on the CaMKII/GluN2B interaction cannot be directly predicted based on our results. Empirically, our findings showed that molecular crowding (100 mg/ml) with BSA, dextran-10 or dextran-70 enhanced binding, while lysozyme and IgG instead decreased it, and PVP-40 showed a small apparent increase that was not statistically significant. An obvious way how a crowding reagent could decrease CaMKII/GluN2B interaction is direct binding to the interaction surface of either protein, but it is difficult to rule out such an effect: A low affinity binding could be reversed during the washes required for the detection assay, but could still cause significant competition at the high concentrations required for molecular crowding. Here, the negative effects on CaMKII/GluN2B binding was correlated with binding of lysozyme and IgG to GST-GluN2B immobilized on a scaffold. While the lysozyme binding was also detected for empty scaffold without GluN2B, even such non-specific scaffold binding could cause steric hindrance for the specific CaMKII/GluN2B interaction. However, other underlying reasons, such as undetected additional direct binding to CaMKII or GluN2B, are also possible.

One reason to test the effect of molecular crowding on the CaMKII/GluN2B interaction was that molecular crowding has been described to affect the CaMKII holoenzyme structure, specifically by favoring a compact closed conformation that inhibits Ca^2+^/CaM binding and that should also make the binding site for GluN2B less accessible [15]. This conclusion regarding the closed holoenzyme conformation was based on the biochemical effects of molecular crowding with 100 mg/ml lysozyme on CaMKII activation by Ca^2+^/CaM [15]. While the unexpected binding of Ca^2+^/CaM to lysozyme observed here provides a direct explanation for the increased Km of Ca^2+^/CaM that was seen in the presence of lysozyme [15], the rationale for the crowding effect on holoenzyme conformation still holds up: While lysozyme increased the apparent Km of Ca^2+^/CaM for both wildtype CaMKII and a mutant impaired for the closed conformation (as expected based on the Ca^2+^/CaM binding to lysozyme observed here), lysozyme enhanced the Hill-coefficient of activation by Ca^2+^/CaM only for wildtype CaMKII but not for this mutant [15]. The model for the increased Ca^2+^/CaM cooperativity is that Ca^2+^/CaM-binding to one kinase subunit increases the likelihood for transition from a closed to an open conformation that facilitates subsequent Ca^2+^/CaM-binding also for the neighboring subunits within a CaMKII holoenzyme. This cooperativity should be significantly higher under conditions that favor the closed conformation, as was indeed observed after molecular crowding. Notably, competition between lysozyme and CaMKII for Ca^2+^/CaM-binding directly explains the observed increase in Km, but should not cause the increase in the Hill-coefficient. Nevertheless, the direct binding to Ca^2+^/CaM found here makes lysozyme a less-than-ideal molecular crowding reagent for future studies on Ca^2+^/CaM-dependent processes.

Another surprise was that our BSA preparation contained sufficient ATP to support Ca^2+^/CaM-stimulated CaMKII autophosphorylation at T286 without the addition of any further ATP, at least when the BSA was added at a high concentration (to a 10% w/v solution, equivalent to 1.5 mM BSA). This contamination may be due to direct binding of ATP to BSA, reported to occur with a Kd of ∼120 µM at pH 7.4 [30], [31]. While BSA could thus also compete with CaMKII for ATP, the affinity of CaMKII for ATP is significantly higher, with a Km that is ∼10-fold lower compared to the Kd for BSA [13], [32]. Additionally, T286 autophosphorylation is among the fastest CaMKII-mediated phosphorylation reactions, with a rate of ∼12^−1^ at 30°C [33]. However, importantly, the enhancement of CaMKII/GlulN2B binding by BSA was not due to CaMKII autophosphorylation, as this enhancement was also seen when autophosphorylation was completely blocked with staurosporine.

It is well described that CaMKII binding to GluN2B is induced by stimulating CaMKII, either directly with Ca^2+^/CaM [9]–[11] or by the Ca^2+^/CaM-dependent autophosphorylation at T286 that generates “autonomous” activity [8], [9], [11]. However, while GluN2B binding is induced by CaMKII stimulation, actual enzymatic CaMKII activity is not required, neither *in vitro* nor within cells [13]. This raised the question if other positive-regulatory factors could also directly induce the CaMKII/GluN2B binding even in absence of stimuli that induce CaMKII activity. Only two other of such positive-regulatory factors are known: nucleotides [12] and the molecular crowding described here. As both of these factors are constitutively present within cells, they could potentially uncouple the induction of CaMKII/GluN2B binding from cellular Ca^2+^-signaling. However, we found that neither macromolecular crowding (with BSA or dextran) nor ADP, neither individually nor combined, was sufficient to induce CaMKII/GluN2B binding without Ca^2+^/CaM (or Ca^2+^/CaM-induced T286 autophosphorylation). Thus, induction of CaMKII binding to GluN2B strictly requires CaMKII stimulation by Ca^2+^-signals, and this requirement is not alleviated by the positive-regulatory effect of the molecular crowding conditions or the high nucleotide concentrations found within cells. Notably, even though nucleotide is not sufficient to induce CaMKII binding to GluN2B, nucleotide binding to CaMKII is required to enable efficient induction of this interaction by Ca^2+^-signals within cells, as was shown using a CaMKII K42M mutant that is incompetent for nucleotide binding [12]. A similar situation may be the case for macromolecular crowding, but this cannot be readily tested experimentally.

In summary, while the nucleotide concentrations and the macromolecular crowding found within cells can enhance CaMKII binding to GluN2B, these factors do not alleviate the requirement for CaMKII stimulation by Ca^2+^-signaling to initiate this binding. Notably, while macromolecular crowding appeared to have positive effect on CaMKII/GluN2B binding in principle, this effect cannot be directly predicted for a specific crowding reagent, as competition of a specific crowding reagent for either binding partner can instead lead to reduced binding.

## Supporting Information

Figure S1 **Effects of increasing concentrations of BSA and lysozyme on CaMKII/GluN2B binding.** Ca^2+^/CaM-stimulated CaMKII binding to GluN2B was tested as in Figure 1A in the presence of the crowding agent BSA or lysozyme (LYS) at six concentrations ranging from 5 mg/ml to 100 mg/ml, with n = 4 for each condition. The graph represents mean ± s.e.m., and GST-GluN2B detection is shown as a loading control.
(PDF)

Figure S2 **Comparison of Ca^2+^/CaM binding to CaMKIIα and lysozyme.** CaMKIIα and lysozyme (2, 1, 0.5, and 0.25 µg) were subjected to SDS-PAGE and transferred to a PVDF membrane. The membrane was first stained for total protein (ponceau, top panel), then incubated with biotin-labeled CaM in the presence of CaCl_2_. Bound CaM was detected by chemi-luminescence. Binding of Ca^2+^/CaM to CaMKIIα was readily detectable at 1 second (middle panel) while binding to lysozyme was not evident until longer exposures (4 minutes, bottom panel).
(PDF)

Figure S3 **Binding of CaMKII to GluN2B in a glutathione-sepharose pull-down assay.** *A*, CaMKIIα (40 nM subunits) binding to GST-GluN2B-C that was immobilized on glutathione-sepharose coated beads was induced by Ca^2+^/CaM (1 mM/1 µM) in the presence of ADP (100 µM) for 15 min at room temperature. Bound CaMKII was eluted and detected by Western-analysis, and quantified by normalized immuno-detection values (IDV). Macromolecular crowding with lysozyme (100 mg/ml) decreased CaMKII binding to GluN2B, while dextran-70 (DEX) (100 mg/ml) increased binding. n = 4; ***: p<0.001 in one-way ANOVA followed by Tukey's HSD. [F(2,11) = 194.16, p<0.001]. Bar graphs indicate mean ± s.e.m, and GST-GluN2B detection is shown as a loading control. The immuno-detection examples are cropped from the same exposure of the same blots. *B*, 100 mg/ml Lysozyme (6.8 mM) or BSA (1.5 mM) were tested for binding to GST-GluN2B-C that was immobilized on glutathione-sepharose coated beads under the same conditions as in Figure 1A. Samples were eluted and subjected to SDS-PAGE, after which the gel was fixed and stained for total protein (silver stain). Lysozyme binding to immobilized GST (without GluN2B fusion), GluN2B, and to empty wells was detected. BSA binding was detected only in the presence of GluN2B. *C*, Ca^2+^/CaM-stimulated CaMKII binding to GluN2B was tested as in Figure 1A. BSA (100 mg/ml) did not significantly affect CaMKII to GluN2B binding, n = 4; [t(3) = 0.694, p = 0.538, two-tailed students t-test.] Bar graphs indicate mean ± s.e.m, and GST-GluN2B detection is shown as a loading control. The immuno-detection examples are cropped from the same exposure of the same blots.
(PDF)

Figure S4 **Calmodulin binding in a blot overlay assay.** Lysozyme, BSA, or IgG (4 µg) were subjected to SDS-PAGE and transferred to a PVDF membrane. The membrane was first stained for total protein (ponceau, right panels), and then incubated with biotin-labeled CaM with or without addition of CaCl_2_. Bound CaM was detected by chemi-luminescence. Ca^2+^/CaM bound to the IgG light chain as well as lysozyme (upper panel). Without the addition of CaCl_2_, at least some CaM binding was detected for all proteins (lower panel), with the signal for lysozyme noticeably weaker compared to binding with CaCl_2_ added.
(PDF)
