# Supplementary material for: CaMKII Binding to GluN2B Is Differentially Affected by Macromolecular Crowding Reagents
Source: PLoS One. 2014 May 5;9(5):e96522. doi: 10.1371/journal.pone.0096522 (PMC4010494; doi:10.1371/journal.pone.0096522)
Supplement: Figure S1 — Effects of increasing concentrations of BSA and lysozyme on CaMKII/GluN2B binding. Ca2+/CaM-stimulated CaMKII binding to GluN2B was tested as in Figure 1A in the presence of the crowding agent BSA or lysozyme (LYS) at six concentrations ranging from 5 mg/ml to 100 mg/ml, with n = 4 for each condition. The graph represents mean ± s.e.m., and GST-GluN2B detection is shown as a loading control. (PDF) [file pone.0096522.s001.pdf]

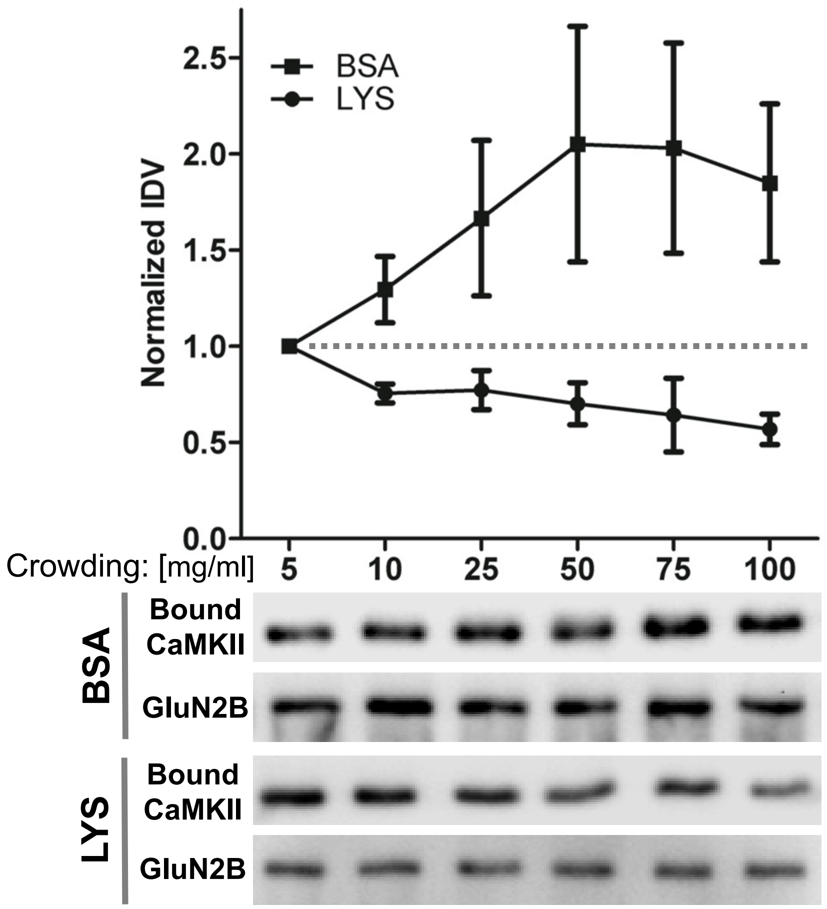

Figure S1. Effects of increasing concentrations of BSA and lysozyme on CaMKII/GluN2B binding.
Ca^{2+}/CaM-stimulated CaMKII binding to GluN2B was tested as in Figure 1A in the presence of the crowding agent BSA or lysozyme (LYS) at six concentrations ranging from 5 mg/ml to 100 mg/ml, with n=4 for each condition. The graph represents mean ± s.e.m., and GST-GluN2B detection is shown as a loading control.
