# Supplementary material for: CaMKII Binding to GluN2B Is Differentially Affected by Macromolecular Crowding Reagents
Source: PLoS One. 2014 May 5;9(5):e96522. doi: 10.1371/journal.pone.0096522 (PMC4010494; doi:10.1371/journal.pone.0096522)
Supplement: Figure S2 — Comparison of Ca2+/CaM binding to CaMKIIα and lysozyme. CaMKIIα and lysozyme (2, 1, 0.5, and 0.25 µg) were subjected to SDS-PAGE and transferred to a PVDF membrane. The membrane was first stained for total protein (ponceau, top panel), then incubated with biotin-labeled CaM in the presence of CaCl2. Bound CaM was detected by chemi-luminescence. Binding of Ca2+/CaM to CaMKIIα was readily detectable at 1 second (middle panel) while binding to lysozyme was not evident until longer exposures (4 minutes, bottom panel). (PDF) [file pone.0096522.s002.pdf]

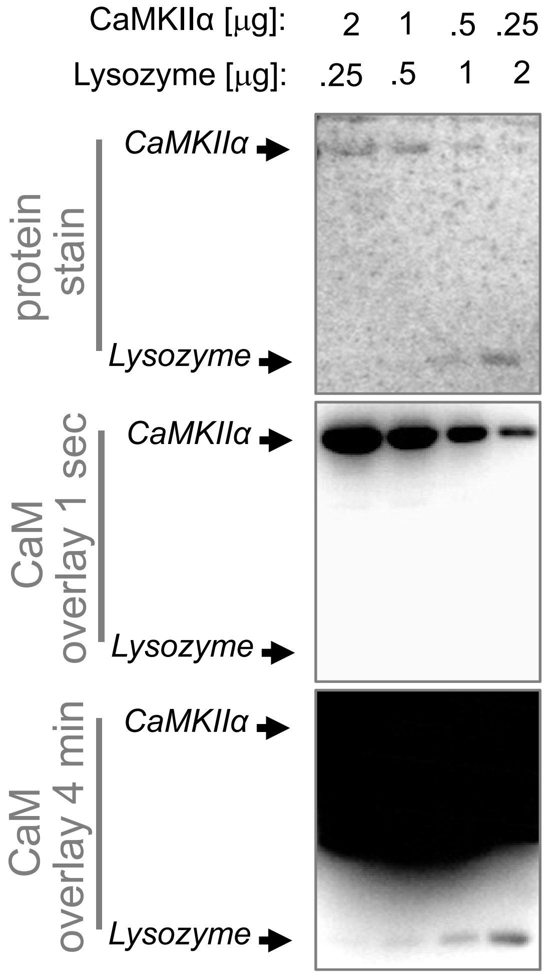

Figure S2. Comparison of Ca^{2+}/CaM binding to CaMKIIα and lysozyme.
CaMKIIα and lysozyme (2, 1, 0.5, and 0.25 μg) were subjected to SDS-PAGE and transferred to a PVDF membrane. The membrane was first stained for total protein (ponceau, top panel), then incubated with biotin-labeled CaM in the presence of $CaCl_2$. Bound CaM was detected by chemi-luminescence. Binding of Ca^{2+}/CaM to CaMKIIα was readily detectable at 1 second (middle panel) while binding to lysozyme was not evident until longer exposures (4 minutes, bottom panel).
