# Supplementary material for: CaMKII Binding to GluN2B Is Differentially Affected by Macromolecular Crowding Reagents
Source: PLoS One. 2014 May 5;9(5):e96522. doi: 10.1371/journal.pone.0096522 (PMC4010494; doi:10.1371/journal.pone.0096522)
Supplement: Figure S3 — Binding of CaMKII to GluN2B in a glutathione-sepharose pull-down assay. A, CaMKIIα (40 nM subunits) binding to GST-GluN2B-C that was immobilized on glutathione-sepharose coated beads was induced by Ca2+/CaM (1 mM/1 µM) in the presence of ADP (100 µM) for 15 min at room temperature. Bound CaMKII was eluted and detected by Western-analysis, and quantified by normalized immuno-detection values (IDV). Macromolecular crowding with lysozyme (100 mg/ml) decreased CaMKII binding to GluN2B, while dextran-70 (DEX) (100 mg/ml) increased binding. n = 4; ***: p<0.001 in one-way ANOVA followed by Tukey's HSD. [F(2,11) = 194.16, p<0.001]. Bar graphs indicate mean ± s.e.m, and GST-GluN2B detection is shown as a loading control. The immuno-detection examples are cropped from the same exposure of the same blots. B, 100 mg/ml Lysozyme (6.8 mM) or BSA (1.5 mM) were tested for binding to GST-GluN2B-C that was immobilized on glutathione-sepharose coated beads under the same conditions as in Figure 1A. Samples were eluted and subjected to SDS-PAGE, after which the gel was fixed and stained for total protein (silver stain). Lysozyme binding to immobilized GST (without GluN2B fusion), GluN2B, and to empty wells was detected. BSA binding was detected only in the presence of GluN2B. C, Ca2+/CaM-stimulated CaMKII binding to GluN2B was tested as in Figure 1A. BSA (100 mg/ml) did not significantly affect CaMKII to GluN2B binding, n = 4; [t(3) = 0.694, p = 0.538, two-tailed students t-test.] Bar graphs indicate mean ± s.e.m, and GST-GluN2B detection is shown as a loading control. The immuno-detection examples are cropped from the same exposure of the same blots. (PDF) [file pone.0096522.s003.pdf]

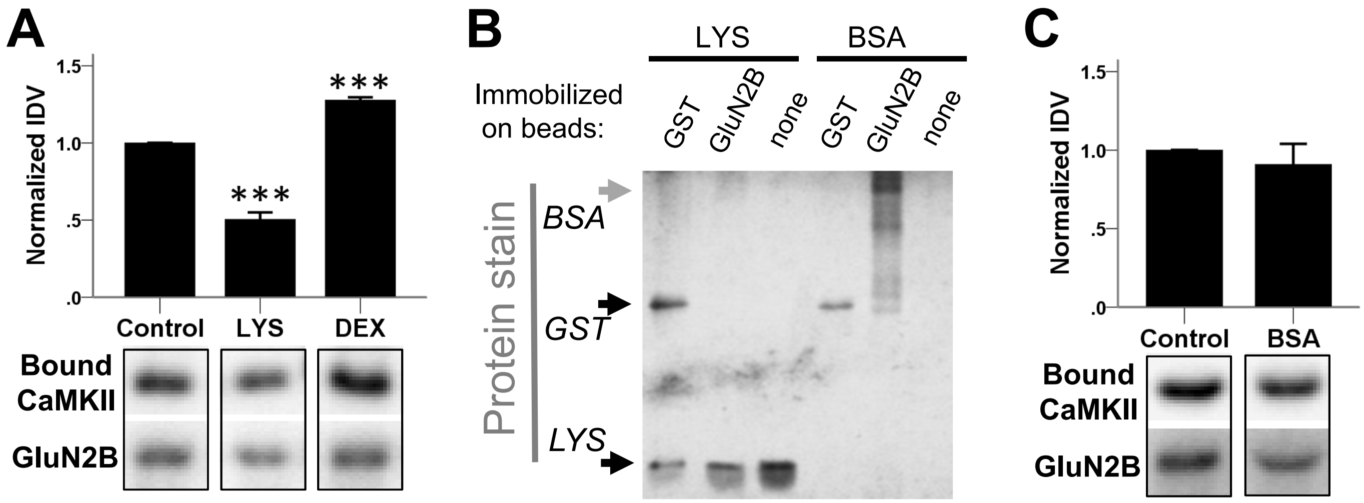

Figure S3. Binding of CaMKII to GluN2B in a glutathione-sepharose pull-down assay.
A, CaMKIIα (40 nM subunits) binding to GST-GluN2B-C that was immobilized on glutathione-sepharose coated beads was induced by Ca^{2+}/CaM (1 mM/1 μM) in the presence of ADP (100 μM) for 15 min at room temperature. Bound CaMKII was eluted and detected by Western-analysis, and quantified by normalized immuno-detection values (IDV). Macromolecular crowding with lysozyme (100 mg/ml) decreased CaMKII binding to GluN2B, while dextran-70 (DEX) (100 mg/ml) increased binding. n=4; ***: p<0.001 in one-way ANOVA followed by Tukey's HSD. [F(2,11)=194.16, p<0.001]. Bar graphs indicate mean ± s.e.m, and GST-GluN2B detection is shown as a loading control. The immuno-detection examples are cropped from the same exposure of the same blots.
B, 100 mg/ml Lysozyme (6.8 mM) or BSA (1.5 mM) were tested for binding to GST-GluN2B-C that was immobilized on glutathione-sepharose coated beads under the same conditions as in Figure 1A. Samples were eluted and subjected to SDS-PAGE, after which the gel was fixed and stained for total protein (silver stain). Lysozyme binding to immobilized GST (without GluN2B fusion), GluN2B, and to empty wells was detected. BSA binding was detected only in the presence of GluN2B.
C, Ca^{2+}/CaM-stimulated CaMKII binding to GluN2B was tested as in Figure 1A. BSA (100 mg/ml) did not significantly affect CaMKII to GluN2B binding, n=4; [t(3)=0.694, p=0.538, two-tailed students t-test.] Bar graphs indicate mean ± s.e.m, and GST-GluN2B detection is shown as a loading control. The immuno-detection examples are cropped from the same exposure of the same blots.
