# Supplementary material for: CaMKII Binding to GluN2B Is Differentially Affected by Macromolecular Crowding Reagents
Source: PLoS One. 2014 May 5;9(5):e96522. doi: 10.1371/journal.pone.0096522 (PMC4010494; doi:10.1371/journal.pone.0096522)
Supplement: Figure S4 — Calmodulin binding in a blot overlay assay. Lysozyme, BSA, or IgG (4 µg) were subjected to SDS-PAGE and transferred to a PVDF membrane. The membrane was first stained for total protein (ponceau, right panels), and then incubated with biotin-labeled CaM with or without addition of CaCl2. Bound CaM was detected by chemi-luminescence. Ca2+/CaM bound to the IgG light chain as well as lysozyme (upper panel). Without the addition of CaCl2, at least some CaM binding was detected for all proteins (lower panel), with the signal for lysozyme noticeably weaker compared to binding with CaCl2 added. (PDF) [file pone.0096522.s004.pdf]

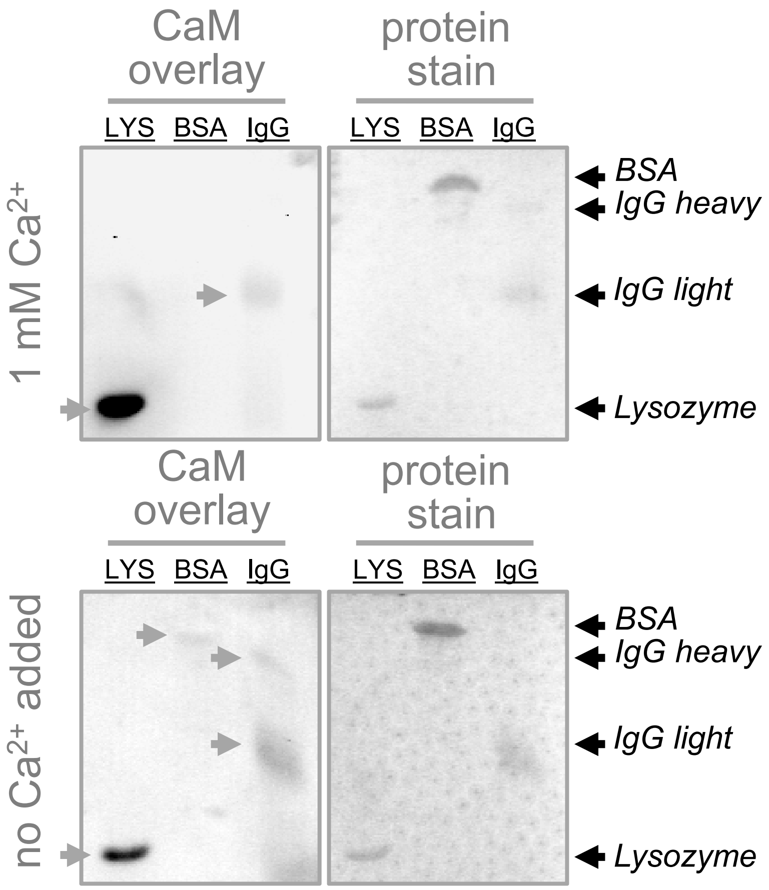

Figure S4. Calmodulin binding in a blot overlay assay. Lysozyme, BSA, or IgG (4 µg) were subjected to SDS-PAGE and transferred to a PVDF membrane. The membrane was first stained for total protein (ponceau, right panels), and then incubated with biotin-labeled CaM with or without addition of $CaCl_2$. Bound CaM was detected by chemi-luminescence. Ca^{2+}/CaM bound to the IgG light chain as well as lysozyme (upper panel). Without the addition of $CaCl_2$, at least some CaM binding was detected for all proteins (lower panel), with the signal for lysozyme noticeably weaker compared to binding with $CaCl_2$ added.
